# Visual Explanation for Identification of the Brain Bases for Developmental Dyslexia on fMRI Data

**DOI:** 10.3389/fncom.2021.594659

**Published:** 2021-09-09

**Authors:** Laura Tomaz Da Silva, Nathalia Bianchini Esper, Duncan D. Ruiz, Felipe Meneguzzi, Augusto Buchweitz

**Affiliations:** ^1^School of Technology, Pontifical Catholic University of Rio Grande do Sul, Porto Alegre, Brazil; ^2^Graduate School of Medicine, Neurosciences, Pontifical Catholic University of Rio Grande do Sul, Porto Alegre, Brazil; ^3^BraIns, Brain Institute of Rio Grande do Sul, Porto Alegre, Brazil; ^4^School of Health and Life Sciences, Psychology, Pontifical Catholic University of Rio Grande do Sul, Porto Alegre, Brazil

**Keywords:** visual explanation, deep learning, dyslexia, neuroimaging, fMRI

## Abstract

**Problem:** Brain imaging studies of mental health and neurodevelopmental disorders have recently included machine learning approaches to identify patients based solely on their brain activation. The goal is to identify brain-related features that generalize from smaller samples of data to larger ones; in the case of neurodevelopmental disorders, finding these patterns can help understand differences in brain function and development that underpin early signs of risk for developmental dyslexia. The success of machine learning classification algorithms on neurofunctional data has been limited to typically homogeneous data sets of few dozens of participants. More recently, larger brain imaging data sets have allowed for deep learning techniques to classify brain states and clinical groups solely from neurofunctional features. Indeed, deep learning techniques can provide helpful tools for classification in healthcare applications, including classification of structural 3D brain images. The adoption of deep learning approaches allows for incremental improvements in classification performance of larger functional brain imaging data sets, but still lacks diagnostic insights about the underlying brain mechanisms associated with disorders; moreover, a related challenge involves providing more clinically-relevant explanations from the neural features that inform classification.

**Methods:** We target this challenge by leveraging two network visualization techniques in convolutional neural network layers responsible for learning high-level features. Using such techniques, we are able to provide meaningful images for expert-backed insights into the condition being classified. We address this challenge using a dataset that includes children diagnosed with developmental dyslexia, and typical reader children.

**Results:** Our results show accurate classification of developmental dyslexia (94.8%) from the brain imaging alone, while providing automatic visualizations of the features involved that match contemporary neuroscientific knowledge (brain regions involved in the reading process for the dyslexic reader group and brain regions associated with strategic control and attention processes for the typical reader group).

**Conclusions:** Our visual explanations of deep learning models turn the accurate yet opaque conclusions from the models into evidence to the condition being studied.

## 1. Introduction

Developmental dyslexia is a neurodevelopmental disorder that presents with persistent difficulty to read fluently and accurately; it is not related to intelligence, lack of educational opportunities or inadequate schooling and affects between 5 and 17% of children (American Psychiatric Association, 2013). Dyslexia is typically diagnosed after about 2–3 years of formal schooling (2nd or 3rd grade), after a child has failed to learn to read. In lower socioeconomic status (SES) countries, studies suggest an even older age at diagnosis among poor children (e.g., 10–11 years; Costa et al., 2015; Buchweitz et al., 2019). However, current neurobiological studies suggest that the functional and anatomical bases associated with dyslexia predate reading instruction (Gabrieli, 2009; Raschle et al., 2014; Ozernov-Palchik and Gaab, 2016; Centanni et al., 2019). In this sense, early identification of developmental dyslexia could help ameliorate the poor mental health and educational outcomes associated with the disorder (Sanfilippo et al., 2020).

There is an emerging consensus about the alterations in brain structure and function associated with dyslexia: functional MRI (fMRI) studies have shown left-lateralized, hypoactivation of posterior brain systems in the ventral occipitotemporal and temporoparietal regions; these regions are part of the brain's network that adapts to reading, in typical readers. The findings of hypoactivation in dyslexia in these posterior brain areas, relative to consistent activation in typical reading, have been replicated across fMRI studies in different languages (Paulesu et al., 2001; Seki et al., 2001; Kronbichler et al., 2006; Cattinelli et al., 2013; Cao et al., 2017; Buchweitz et al., 2019). On the other hand, a typical reader usually shows consistent activation of these occipitotemporal and parietotemporal posterior brain systems; these regions become functionally and morphologically integrated with the areas of the brain that are hardwired for spoken language as one learns to read (Pugh et al., 1996; Michael et al., 2001; Shaywitz et al., 2004; Buchweitz et al., 2009; Rueckl et al., 2015). The adaptations of these posterior brain regions represent brain markers of reading development, and their hypoactivation and altered function, markers of dyslexia. As markers of risk for dyslexia, understanding how these regions function and adapt can potentially inform earlier identification of risk for dyslexia and better understanding of reading treatment response (Gabrieli, 2009; Van Den Bunt et al., 2018).

Distinct brain imaging techniques such as structural MRI, fMRI, and diffusion-weighted imaging (DWI) are applied to investigate altered cortical tissue, structure and function associated with mental health and neurodevelopmental disorders (Atluri et al., 2013). These techniques allow for the identification of neural markers, which in turn may provide or inform a diagnosis based on image features (American Psychiatric Association, 2013).

Recent advances in deep learning have led researchers to employ machine learning to automate the analysis of medical imaging, including neurological images (Craddock et al., 2009; Froehlich et al., 2014; Tamboer et al., 2016). The most successful technique derived from deep learning for image classification consists of building neural network with convolutional layers, i.e., Convolutional Neural Networks (CNNs). The CNN specializes in processing multiple arrays, such as images (2D), audio and video or volumetric data (3D) (Bengio et al., 2015).

Brain imaging volumes have tens of thousands of voxels (3D-pixel) per image. Neurofunctional indices are mapped to these voxels, which makes feature selection a challenge for most machine learning approaches. Supervised approaches to machine learning relied on experts for feature selection (Bengio et al., 2015). Deep learning approaches obviate the dependence on supervision by automatically learning the features that better represent the problem domain (Bengio et al., 2015). Before deep learning methods were effectively applied to classification of brain imaging data, support vector machine (SVM) algorithms were the frequent choice for machine learning analyses of brain imaging (Cortes and Vapnik, 1995). SVM algorithms have the ability to generalize well in smaller fMRI datasets (Craddock et al., 2009; Buchweitz et al., 2012; Froehlich et al., 2014; Li et al., 2014; Tamboer et al., 2016; Just et al., 2017), which are typically in the dozens of participants due to the high costs of fMRI scans (Craddock et al., 2009; Froehlich et al., 2014). Moreover, SVM models trained with linear kernels offer relatively straightforward explanations. This SVM characteristic may be useful to break the “curse of dimensionality” by reducing the risk of overfitting the training data. The number of voxels used in feature selection should be reduced as much as possible.

Feature selection for brain imaging data is often performed on voxels in anatomically or functionally defined regions-of-interest (ROIs) based on the literature (Wolfers et al., 2015) or by data-driven methods that establish clusters of stable voxels (Shinkareva et al., 2008; Just et al., 2014). By contrast, deep learning models learn feature hierarchies at several levels of abstraction, which allows the system to learn complex functions independent of human-crafted features (Bengio et al., 2015). CNNs are applicable to a variety of medical image analysis problems, such as disorder classification (Heinsfeld et al., 2018), anatomy or tumor segmentation (Kamnitsas et al., 2017), lesion detection and classification (Ghafoorian et al., 2017), survival prediction (van der Burgh et al., 2017), and medical image construction (Li et al., 2014). Although these models can be accurate, their conclusions are opaque to human understanding and lack a straightforward explanation to help diagnosis. The provision of tools for healthcare practitioners to apply and trust the results of machine learning models of brain imaging to assist them in their clinical diagnoses is a challenge for brain imaging and machine learning research alike. Providing accurate visual representation of neural networks involved in deep learning classification may be a step in the direction of improving diagnostic application of classification using neurofunctional indices. For example, the prediction of brain states at slice level, and the subsequent generation of more fine-grained information about the features relevant for classification, can help improve interpretability (Ballester et al., 2021).

The goal of the present study is to integrate feature visualization techniques for CNNs. The key contribution is a visual representation of the regions involved in classifying whether children are dyslexic or not. This provides a better understanding of CNN behavior and may provide practitioners with a tool to glean neural alterations associated with a disorder from functional brain imaging scans.

## 2. Materials and Methods

### 2.1. Data

The brain imaging data was collected as part of a research initiative to investigate the neural underpinnings of dyslexic children in Brazil. The participants were diagnosed with dyslexia following a multidisciplinary evaluation that included medical history, reading and writing tests (Costa et al., 2015; Toazza et al., 2017), and an IQ test (Wechsler Abbreviated Scale of Intelligence, Wechsler, 2012). The reading and other tests applied are described elsewhere (Costa et al., 2015; Buchweitz et al., 2019); in the interest of providing comprehensive information about the participants, see also the Supplementary Materials (supplementary information about participants and instruments).

#### 2.1.1. Participants

The present study included 32 children who were divided into two groups: typical readers (TYP; *n* = 16) and dyslexic readers (DYS; *n* = 16) (Buchweitz et al., 2019). The participants were all monolingual speakers of Portuguese and right-handed. The two groups were matched for age, sex and IQ [age 8−12 (9 ± 1.39)]. The typical readers were scanned during the 2015 school year; the dyslexic children were scanned between 2014 and 2015 (Buchweitz et al., 2019). Table 1 summarizes the complete demographics on this dataset. As indicate above, see also the Supplementary Materials for additional information on participants.

**Table 1:** Demographic information of the study subjects.

	**Typical readers**	**Dyslexic readers**	***p*-value^***a***^**
No. of subjects	16	16	
Age			
Mean ± STD	8.44 ± 0.51	9.63 ± 0.88	<0.001
Range	8–9	8–12	
Sex (Male/Female)	9 / 7	11 / 5	
IQ			
Mean ± STD	102.73 ± 15.37	107.85 ± 26.6	NS
Range	71–127	88–144	
Socioeconomic status (SES) (mean ± STD)	24.1 ± 4.9	26.4 ± 6.5	
Reading speed—words per minute (mean ± STD)	84.71 ± 31.89	13.07 ± 7.68	<0.001
Average motion—fMRI Task (mean ± STD)	0.17 ± 0.15	0.26 ± 0.08	NS
fMRI task—accuracy (mean ± STD)	54.68 ± 6.71	35.06 ± 14	<0.001
fMRI task—response time (mean ± STD)	2039.2 ± 423.56	2981.06 ± 954.82	NS

a *Independent samples t-test; STD, standard deviation; NS, not significant*.

#### 2.1.2. Word-Reading Task

Task based fMRI examines brain regions whose activity changes from baseline in response to the performance of a task or stimulus (Petersen and Dubis, 2012). The study was designed as a mixed event-related experiment using a word and pseudoword reading test validated for Brazilian children (Salles et al., 2013). The task consisted of 20 regular words, 20 irregular words, and 20 pseudowords. The 60 stimuli were divided into two 30-item runs to give the participants a break halfway into the task. Words and pseudowords were presented on the screen one at a time for 7 s each. A question was presented to participants along with each word (“Does the word exist?”), to which participants had to select “Yes” or “No” by pressing response buttons. After 10 trials (10 words) either a baseline condition or rest period was inserted in the experimental paradigm. The baseline condition consisted of presentation of a plus sign “+” in the middle of the screen for 30 s, during which participants were instructed to relax and clear their minds (Buchweitz et al., 2019).

#### 2.1.3. Data Acquisition

Data was collected on a GE HDxT 3.0 T MRI scanner with an 8-channel head coil (Buchweitz et al., 2019). The following MRI sequences were acquired: a T1 structural scan (TR/TE = 6.16/2.18 ms, isotropic 1 mm^3^ voxels); two task-related 5-min 26-s functional fMRI EPI sequences; and a 7-min resting state sequence. The task and the resting-state EPI sequences used the following parameters: TR = 2,000 ms, TE = 30 ms, 29 interleaved slices, slice thickness = 3.5 mm; slice gap = 0.1 mm; matrix size = 64 × 64, FOV = 220 × 220 mm, voxel size = 3.44 × 3.44 × 3.60 mm (Buchweitz et al., 2019).

#### 2.1.4. Data Preprocessing

The preprocessing steps for the task-based (word-reading task) fMRI are described as follows. Word-reading task: preprocessing included slice-time and motion correction, smoothing with a 6 mm FWHM Gaussian kernel, and a nonlinear spatial normalization to 3.0 × 3.0 × 3.0 mm voxel template (HaskinsPedsNL template). TRs with motion outliers (>0.9 mm) were censored from the data. The criteria for exclusion due to head motion were: excessive motion in 20% of the TRs. The average head motion for each group for the participants included in the study, in the word-reading paradigm, was: DYS M = 0.16 ± 0.08, TYP M = 0.18 ± 0.15 (Buchweitz et al., 2019).

First level analysis included modeling regressor for the conditions for each of the three types of word (regular words, irregular words and pseudowords), and for the fixation condition. As a final preprocessing step, we averaged the words activation, and used this average as an input to the deep learning models. **T**-test analysis (3*dttest*++) were carried out to compare the distribution of activation between the two groups using a random-effects model and the contrast images for all the word types vs. fixation. Participant age was entered as a covariate in the analysis between groups to control for any effects due to the average 1-year difference in age between the groups. Table 1 shows the demographics for the dataset used in this study. The accuracy and response time, during the MRI exam, were statistically significant between groups.

### 2.2. Classification Task

We trained a number of deep learning models for the classification task using two key recent techniques in learning for image classification: CNNs (LeCun et al., 1998) and data augmentation (Perez and Wang, 2017). For the CNNs, we evaluated both two-dimensional (2D) and three-dimensional (3D) CNNs.

**Figure 1:**
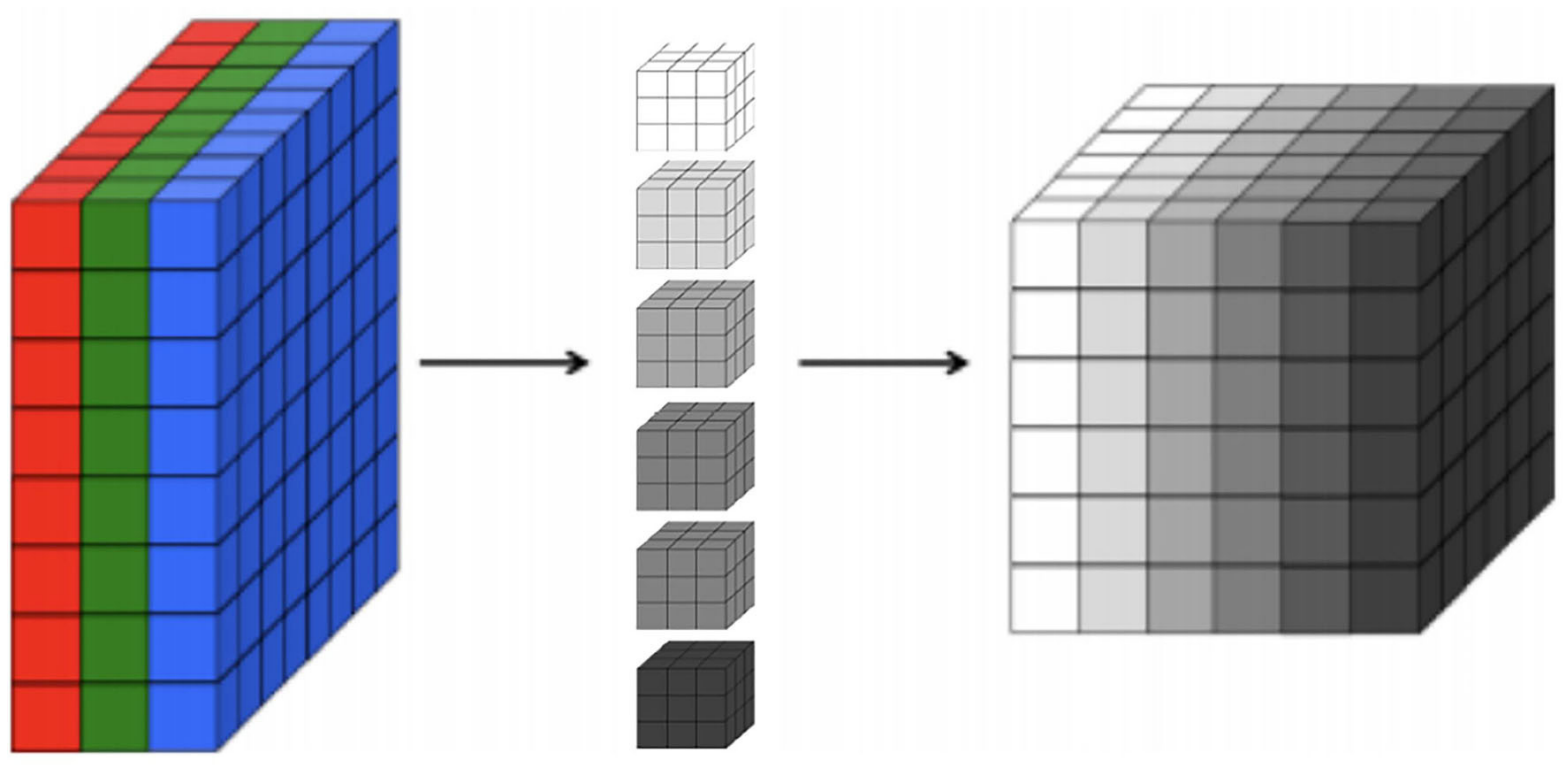
A 3D representation of a convolutional layer, where each RGB channel in the input is a colored slice. We show six filters with the same depth of the input in the middle and, on the right, we show the output activations of combined convolution operations where each slice in the output corresponds to each filter.

First, regarding the CNNs, our choice of model focuses on 2D CNNs due to the size of our dataset. Specifically, 2D CNNs have a smaller number of parameters in comparison to 3D CNNs (Szegedy et al., 2015). Thus, training a 3D CNN necessitates substantially larger datasets in order to generalize well. Indeed, our experiments show that 2D CNNs achieve superior accuracy to 3D CNNs given the limitations of our dataset. A 2D CNN takes an input having three dimensions (a height *h*, a width *w*, and a number of color channels or a depth *d*). This input volume is then processed by *k* filters, which operate on the entire volume of feature maps that have been generated at a particular layer. 2D convolutions have a pseudo third dimension comprising the color channels in each image, such that a 2D CNN applies convolutions to each channel separately, combining the resulting activations. Figure 1 illustrates each RGB channel in the input as a slice. A filter, which corresponds to weights in the convolutional layer, is then multiplied with a local portion of the input to produce a neuron in the next volumetric layer of neurons. In the Figure 1, the middle part represents filters, the depth of the filter corresponds to the depth of the input. The last cube in the figure represents the output activations of the combined convolution operations for each channel. The depth of the output volume of a convolutional layer is equivalent to the number of filters in that layer, that is, each filter produces its own slice. This can be viewed as using a 3D convolution for each output channel, which happens to have the same depth as the input (Buduma and Locascio, 2017). For this reason, it is possible to use volumetric images as inputs to a 2D CNN. In effect, this means that a 2D CNN processes the 3D volume of brain scan activations slice-by-slice.

Second, we avoid overfitting in our small dataset by employing data augmentation. Data augmentation is a technique (Perez and Wang, 2017) that provides the model with more data to increase the model's ability to generalize from it. Such techniques are already employed in several image problems in deep learning models, but are still incipient in fMRI data (Mikołajczyk and Grochowski, 2018).

We adopted two approaches to build the 2D CNN architectures: (i) use genetic programming, more specifically grammar-based genetic programming (GGP) fitted to our problem; and (ii) employ a modified version of the LeNet-5 (LeCun et al., 1998) classification model. We then trained the resulting architecture using our dataset, and compared the effectiveness of 3D convolutions by converting the generated 2D CNNs into 3D ones by swapping the 2D convolutional layers to appropriately-sized 3D convolutions.

### 2.3. Visual Explanations Task

While many application areas for machine learning focus simply on model performance, recent work has highlighted the need for explanations for the decisions of trained models. Most users of machine learning often want to understand the trained models in order to gain confidence in the predictions. This is especially true for machine learning models used in medical applications, where the consequences of each decision must be carefully explained to patients and other stakeholders (Yang et al., 2018; Jin et al., 2019). Besides the explainability aspect required of direct medical applications, our key motivation is to allow neuroimaging specialists to derive new insights on underpinnings of specific learning disorders such as dyslexia. Indeed, clinical diagnosis of dyslexia is reliable and costs less than using fMRI scans to validate such diagnostics (Torgesen, 1998; Ramus et al., 2003). However, researchers of dyslexia are interested in further understanding of the disorder and its neural underpinnings *in-vivo* (Shaywitz et al., 2001; Hoeft et al., 2011). For this reason, building data-driven diagnostics models via machine learning and generating explanations for such models can be an invaluable tool for dyslexia research.

Recently researchers developed several methods for understanding and visualizing CNNs, in part as a response to criticism that the learned features in a neural network are not interpretable to humans (Szegedy et al., 2014; Zeiler and Fergus, 2013; Zhou et al., 2016). A category of techniques that aim to help understand which parts of an image a CNN model uses to infer class labels is called Class Activation Mapping (CAM) (Zhou et al., 2016). CAM produces heatmaps of class activations over input images. A class activation heatmap is a 2D grid of scores associated with a particular output class, computed for every location for an input image, indicating how important each location is with respect to that output class (Zhou et al., 2016). CAM can be used by a restricted class of image classification CNNs, precluding the model from containing any fully-connected layers and employing global average pooling (GAP).

A recent approach to visualize features learned by a CNN is Grad-CAM (Selvaraju et al., 2017). Grad-CAM is a generalization of CAM and can be applied to a broader range of CNN models without the need to change their architecture. Instead of trying to propagate back the gradients, Grad-CAM infers a downsampled relevance heatmap of the input pixels from the activation heatmaps of the final convolutional layer. The downsampled heatmap is upsampled to obtain a coarse relevance heatmap. This approach has two key advantages: first, it can be applied to any CNN architecture; and second, it requires no re-training or change in the existing neural network architecture.

**Figure 2:**
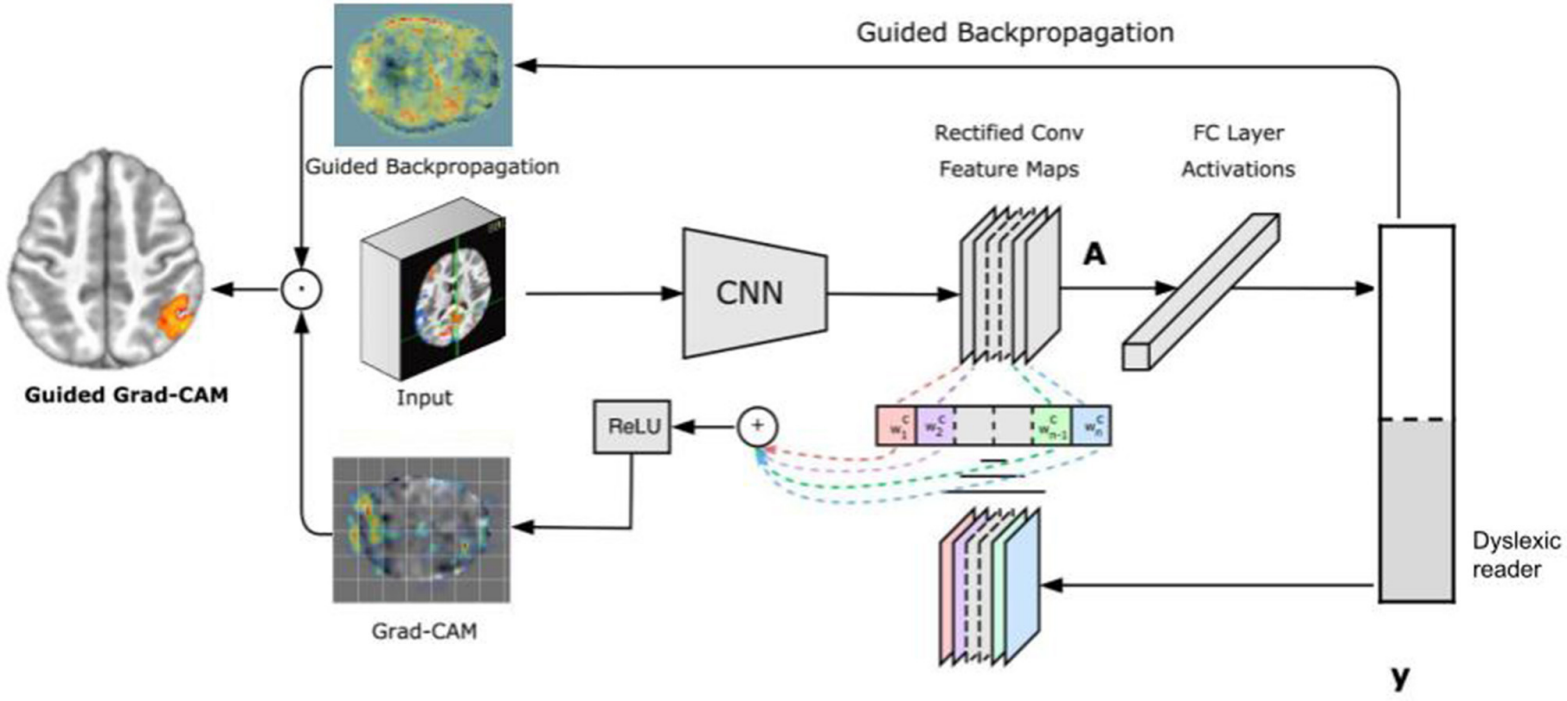
Grad-CAM overview.

Figure 2 illustrates the Grad-CAM approach. Given an image and a class of interest (in the example, “dyslexic reader”) as input, Grad-CAM first forward propagates the image through the CNN part of the model and then through task-specific computations to obtain a raw score for the category. Next, Grad-CAM sets all the gradients that do not belong to the desired class (dyslexic reader), which are originally set to one, are set to zero. Grad-CAM then backpropagates this signal to the rectified convolutional feature maps of interest, which it combines to compute the coarse Grad-CAM localization (the bottom heatmap in the figure) representing where the model has to look to make the particular decision. Finally, Grad-CAM pointwise multiplies the heatmap with guided backpropagation to get Guided Grad-CAM visualizations which are both high-resolution and concept-specific (Selvaraju et al., 2017).

## 3. Experiments and Results

### 3.1. Classification

The deep learning classification model was implemented using the Keras open source library (Chollet et al., 2015) and trained with an Nvidia Geforce GTX 1080 Ti graphical processing unit (GPU) with 12 GB of memory. In our GGP approach, we generated a population of CNN architectures, such that each CNN architecture was an individual in a population, and was evaluated to produce a fitness value. Network topology for all CNNs generated was based on a specific grammar for our problem and a set of different hyperparameters.

**Figure 3:**
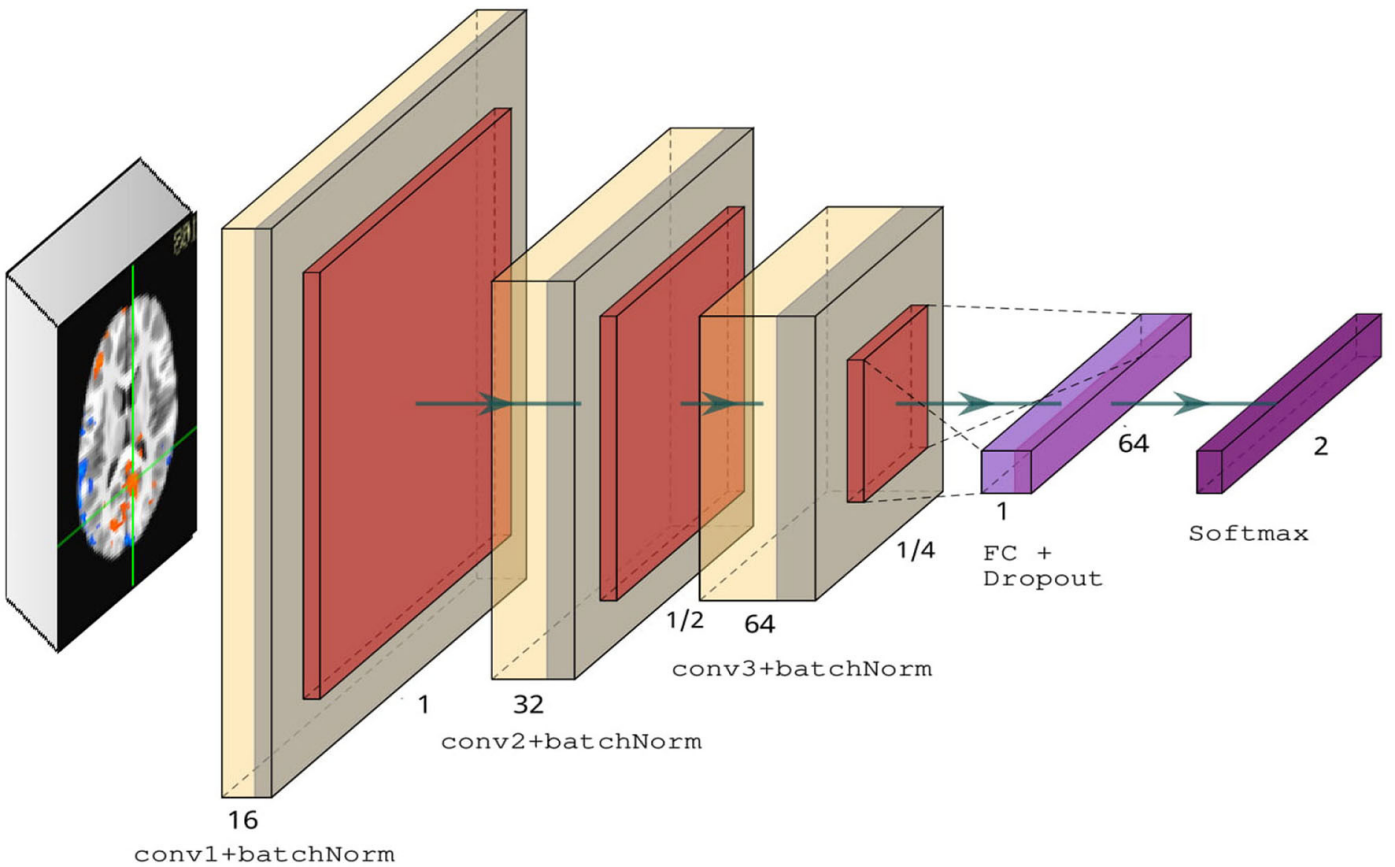
Modified LeNet-5 overview containing three convolutional layers with ReLU activations, followed by a fully connected layer and dropout, and finally a softmax classifier.

We introduced four key modifications in our version of the LeNet-5 architecture. First, we added batch normalization layers in the convolutional layers to improve convergence and generalization (Ioffe and Szegedy, 2015). Second, we used ReLU activations in the convolutional layers instead of tanh. Third, we changed the average pooling to max pooling in the subsampling layers. Finally, we used a dropout rate of 0.5 in the fully connected layer. Figure 3 illustrates our modified version of LeNet-5. Our model architecture contains ~175 K parameters, a small amount in comparison to deeper architectures, such as VGG-16 (Simonyan and Zisserman, 2014), which contains over 138 million parameters.

Our 3D CNN was developed based on our 2D CNN model. We made the changes necessary to adapt 2D convolutions, 2D pooling layers to a 3D model. In order to fit our data to a 3D CNN model, we expanded our data adding one channel for gray images resulting in a 4-dimensional array as input to the network. The resulting architecture has over 3 million parameters.

We compared our induced deep learning models with the SVM (Cortes and Vapnik, 1995) technique, which has been used in a substantial number of previous neuroimaging studies (Froehlich et al., 2014; Tamboer et al., 2016). Specifically, this technique is popular for fMRI applications because datasets typically have many features (voxels), but only a relatively small set of subjects.

We trained all models to classify the participants between dyslexic readers and typical readers using the Adam optimizer (Kingma and Ba, 2014). We improved the performance of our classifier by employing two data augmentations to our dataset: (i) we added Gaussian noise to fMRI images to generalize to noisy images; and (ii) we added a random Gaussian offset, or contrast, to increase differences between images. The input of our machine and deep learning models was the whole brain volume (60 × 73 × 60 voxels) and a binary mask filling the brain volume to retrieve data from all brain regions. All of our deep learning models followed the same split, i.e., 80% train, 10% validation, and 10% test sets. The parameter values including learning rate, dropout rate, batch size, and epoch size were optimized using the ranges summarized in Table 2. Note that we optimized the batch size to use the maximum available GPU memory.

**Table 2:** CNN hyperparameters used to generate our GGP population of CNN architectures.

**Hyperparameters**	**Values**
Kernel size	Ranging from 1 to 5
No. of filters	Starts with 16; duplicates after every convolution
Stride	Ranging from 1 to 3
Learning rate	Logarithmic range of [1, 0.1, 0.01, 0.001, 0.0001, 0.00001]
Dropout rate	Tuned in the range of [0.1, 0.5, 1]
Batch size	16
No. of epochs	Tuned in the range of [10, 50, 100]
No. of Neurons FC layer	Tuned in the range of [32, 64, 128, 256, 512]

All hyperparameters were optimized for both the 2D and 3D CNN models. For our SVM models, first, we applied an exhaustive search over specified parameters values for our SVM estimator. Second, we evaluated different methods of cross-validation. We report the results from splitting the data into train, validation, and test for Linear SVM implemented using scikit-learn (Pedregosa et al., 2011) library in Python.

Our modified version of LeNet-5 2D CNN network achieved 85.71% accuracy on subject classification. Our best GGP 2D CNN model achieved an accuracy of 94.83% on subject classification. In comparison to the 2D CNN architecture, the 3D CNN, from both the modified LeNet-5 and GGP approach, had an inferior accuracy on subject classification. The 3D CNN was also more prone to overfitting in the first few epochs of training. By contrast, the SVM approach achieved much lower classification accuracy, regardless of the training dataset composition. Table 3 summarizes the results from all our classification approaches.

**Table 3:** Summary of dyslexia classification results, including our Modified LeNet-5 architecture (2D and 3D) and our best GGP CNN.

**Technique**	**Accuracy (%)**
**Best GGP 2D CNN**	**94.83**
Modified LeNet-5	85.71
Best GGP 3D CNN	78.57
Modified LeNet-5 3D	71.43
SVM (80% train, 20% test)	70

### 3.2. Visual Explanations

After training the 2D CNN model, we loaded the model with the best accuracy to visualize the learned gradients using Grad-CAM technique (Selvaraju et al., 2017). The class activation generated by Grad-CAM shows which regions were more instrumental to the classification.

**Figure 4:**
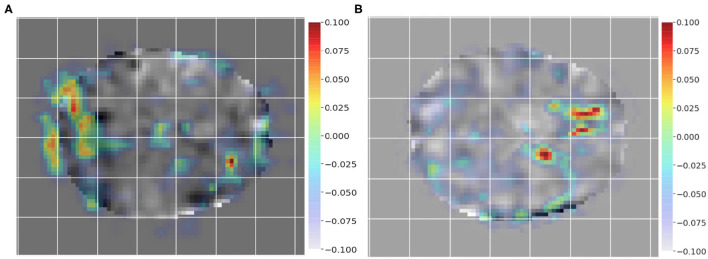
Class activation mapping for a single dyslexic reader **(A)** and single typical reader **(B)** subject classification from Grad-CAM technique. The visualization highlights areas with lower class activation colored from gray to light blue, whereas areas with higher class activation are colored from yellow to red.

To employ Grad-CAM visualization to identify key differences between subjects and controls, we chose a pair of subjects as input, i.e., a control (non-dyslexic) subject and a dyslexic reader subject to generate the class activation mappings. Figures 4A,B show Grad-CAM generated images of control and dyslexic readers subjects, with respect to the gradients learned by the network model. Both images depict the central slice from the axial view of the brain volume. Areas with lower class activation mappings are colored in gray, whereas areas with higher class activation mappings are color-coded from yellow (instrumental) to red (more instrumental). The color coding thus represents the brain regions impact on the model classification of subjects.

**Figure 5:**
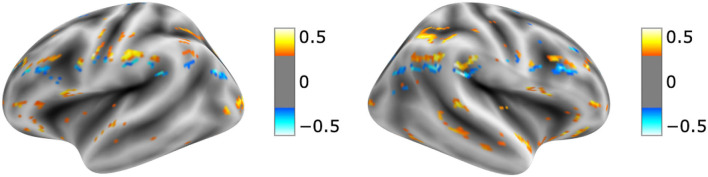
Visual explanation for Dyslexic readers subjects. Supplementary Material contains axial images, instrumental brain regions for dyslexic readers identification summarized in Table 4. The left side of the images represents the left side of the brain. Surface images for left and right side of the brain showing the visual explanations at cortical level. AFNI (Cox, 1996) images showing brain activation from Grad-CAM.

**Figure 6:**
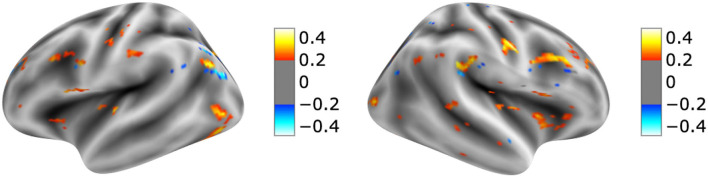
Visual explanation for Typical readers subjects. Supplementary Material contains axial images, instrumental brain regions for Typical readers identification summarized in Table 5. The left side of the images represents the left side of the brain. Surface images for left and right side of the brain showing the visual explanations at cortical level. AFNI (Cox, 1996) images showing brain activation from Grad-CAM.

The visualization for the dyslexic readers group (Figure 5) showed frontal and temporal brain regions that are traditionally associated with reading processes, and also temporoparietal and dorsolateral prefrontal regions that are associated with increased working memory load, including during reading (Pugh et al., 1996; Chein and Schneider, 2005; Buchweitz et al., 2009, 2014; Waldie et al., 2013). Additional axial visualizations of brain regions can be found in Supplementary Figure 2 showed bilateral inferior frontal gyrus (Supplementary Figures 2A,C,D), the parietal lobe (Supplementary Figure 2F) and the right temporal lobe (Supplementary Figure 2E) were some of the regions that presented high classification mapping in the group analysis. In addition to the frontal regions, the group analysis (Figure 6) showed that the left precuneus (Supplementary Figure 3B) and the right insula (Supplementary Figure 3D) were also among the regions with higher classification mapping for typical readers relative to dyslexic readers (Oh et al., 2014). Tables 4, 5 show the voxel count per brain region for visualization of the dyslexic readers group and for the typical readers group, respectively. For group-level analyses of brain activation differences between dyslexic readers and typical readers, please see Buchweitz et al. (2019), which included the same participants.

**Table 4:** Voxel count per brain region of dyslexic readers for Supplementary Figure 2.

**Dyslexic readers**			**Peak of activation coordinates**		
	**Brain regions**	**No. of voxels**	**x**	**y**	**z**
Supplementary Figure 2A	**Left inferior frontal gyrus**	**167**	**−40**	**27**	**27**
	*Left rostral middle frontal*	52			
	*Left IFG^*a*^ (pars opercularis)*	20			
	*Left postcentral*	18			
	*Left precentral*	18			
	*Left superior frontal*	13			
	*Left supramarginal*	5			
	*Left caudal middle frontal*	5			
	*White matter*	36			
Supplementary Figure 2B	**Left superior frontal gyrus**	**123**	**−11**	**48**	**24**
	*Left rostral middle frontal*	43			
	*Left IFG (pars opercularis)*	23			
	*Left superior frontal*	19			
	*Right superior frontal*	9			
	*Left precentral*	4			
	*Left caudal middle frontal*	3			
	*Right rostral middle frontal*	3			
	*Left caudal anterior cingulate*	1			
	*White matter*	18			
Supplementary Figure 2C	**Right IFG (pars opercularis)**	**116**	**52**	**9**	**24**
	*Right IFG (pars opercularis)*	40			
	*Right rostral middle frontal*	21			
	*Right precentral*	14			
	*Right postcentral*	4			
	*Right caudal anterior cingulate*	2			
	*Right IFG (pars triangularis)*	1			
	*White matter*	34			
Supplementary Figure 2D	**Right IFG (pars triangularis)**	**98**	**49**	**24**	**30**
	*Right rostral middle frontal*	33			
	*Right IFG (pars opercularis)*	14			
	*Right caudal middle frontal*	12			
	*Right precentral*	11			
	*Right postcentral*	2			
	*White matter*	26			
Supplementary Figure 2E	**Right middle temporal**	**77**	**61**	**-8**	**-15**
	*Right inferior temporal*	30			
	*Right middle temporal*	22			
	*Right superior parietal*	11			
	*White matter*	14			
Supplementary Figure 2F	**Right angular**	**65**	**46**	**−57**	**45**
	*Right inferior parietal*	59			
	*Right supramarginal*	4			
	*White matter*	2			

*Brain regions instrumental for dyslexic readers identification with Grad-CAM (Selvaraju et al., 2017). Region labels follow Haskins pediatric atlas (Molfese et al., 2020)*.

a *IFG, inferior frontal gyrus*.

**Table 5:** Voxel count per brain region of typical readers for Supplementary Figure 3.

**Typical readers**			**Peak of activation coordinates**		
	**Brain regions**	**No. of voxels**	**x**	**y**	**z**
Supplementary Figure 3A	**Right postcentral**	**201**	**43**	**−11**	**30**
	*Right supramarginal*	43			
	*Right IFG^*a*^ (pars opercularis)*	29			
	*Right caudal middle frontal*	25			
	*Right postcentral*	17			
	*Right precentral*	14			
	*Right supramarginal*	8			
	*Right inferior parietal*	7			
	*White matter*	58			
Supplementary Figure 3B	**Left precuneus**	**89**	**−1**	**−68**	**39**
	*Left precuneus*	35			
	*Left inferior parietal*	25			
	*Right precuneus*	10			
	*Left superior parietal*	7			
	*Left cuneus*	2			
	*Right cuneus*	1			
	*White matter*	9			
Supplementary Figure 3C	**Left superior occipital**	**82**	**−16**	**-89**	**24**
	*Left inferior parietal*	51			
	*Left lateral occipital*	17			
	*Left cuneus*	7			
	*White matter*	7			
Supplementary Figure 3D	**Right insula**	**64**	**31**	**−23**	**-24**
	*Right supramarginal*	19			
	*Right postcentral*	9			
	*Right insula*	2			
	*Right caudate*	2			
	*White matter*	32			

*Brain regions instrumental for typical readers identification with Grad-CAM (Selvaraju et al., 2017). Region labels follow Haskins pediatric atlas (Molfese et al., 2020)*.

a *IFG, inferior frontal gyrus*.

## 4. Discussion and Related Work

To the best of our knowledge, there is little work on visual explanations and brain imaging; for instance, a recent study used these explanations for Alzheimer's disease (AD) and structural MRI (sMRI) (Jin et al., 2019). However, few approaches employed a visualization technique for MRI data, and there are none for fMRI data. The lack of approaches using brain imaging data of Dyslexia led us to search for related work employing deep learning to process any type of MRI data. Table 6 summarizes previous work that employed deep learning (Sarraf and Tofighi, 2016; Heinsfeld et al., 2018; Jin et al., 2019) for subject classification, and approaches that applied machine learning to identify participants with dyslexia (Cui et al., 2016; Tamboer et al., 2016; Płoński et al., 2017).

**Table 6:** Comparison with the classification scores of related work.

**Study references**	**Modality**	**Dataset**	**Classifier**	**Task**	**Accuracy (%)**
Proposed method	Task based fMRI	ACERTA project	2D CNN	Subject classification for Dyslexia	94.83
Sarraf and Tofighi (2016)	rs-fMRI	ADNI^*a*^	LeNet-5	Subject classification for Alzheimer	96.86
Jin et al. (2019)	sMRI	ADNI^*a*^	Attention-based 3D ResNet	Subject classification for Alzheimer's Disease	92.1%
Cui et al. (2016)	sMRI	Private dataset	SVM	Subject classification for Dyslexia	83.6
Tamboer et al. (2016)	sMRI	Non-disclosed dataset	SVM	Subject classification for Dyslexia	80
Heinsfeld et al. (2018)	rs-fMRI	ABIDE	Denoising Autoencoder	Subject classification for Autism Spectrum Disorder	Above 70
Płoński et al. (2017)	sMRI	Private dataset	SVM, LR, RF	Subject classification for Dyslexia	65

a *adni.loni.usc.edu*.

The machine learning techniques we use in this article allow us to divide the related work into two types: (i) work that aimed to identify participants with dyslexia using traditional machine learning algorithms (e.g., SVM); and (ii) work that used Deep Neural Networks (DNNs) in brain imaging data for disease classification, as follows. Sarraf and Tofighi (2016) employed the LeNet-5 architecture to classify patients with Alzheimer's disease. Heinsfeld et al. (2018) used two stacked denoising autoencoders for the unsupervised pre-training stage to extract a lower-dimensional version of the ABIDE (Autism Brain Imaging Data Exchange) data. Jin et al. (2019) employed an attention-based 3D residual network based on the 3D ResNet to classify Alzheimer's Disease classification and to identify important regions in their visual explanation task. The remaining work applied machine learning techniques to classify dyslexic readers and typical readers subjects. Tamboer et al. (2016) and Cui et al. (2016) used SVM. Płoński et al. (2017) on top of using SVM, also used logistic regression (LR), and random forest (RF).

Approaches that adopt deep learning models (Sarraf and Tofighi, 2016; Heinsfeld et al., 2018; Jin et al., 2019) show that DNN approaches can achieve competitive results using MRI and fMRI data. Heinsfeld et al. (2018) achieved state-of-the-art results with 70% accuracy in identification of ASD vs. control patients in the dataset. The authors that used classic machine learning techniques (Cui et al., 2016; Tamboer et al., 2016; Płoński et al., 2017) achieved 80, 83.6, and 65% accuracy respectively on dyslexia prediction from anatomical scans. Performance of our deep learning models was consistent with other deep learning approaches for classification of neurological conditions. By contrast, our SVM results did not generalize as well as others (Cui et al., 2016; Tamboer et al., 2016; Płoński et al., 2017), but still outperformed another application of SVM for dyslexia classification (Płoński et al., 2017). Given the difference in datasets, accuracies obtained by our two approaches are not comparable to other ones.

Jin et al. (2019) visual explanations consisted of an attention map (much like a heatmap in visual representation) that indicated the significance of brain regions for AD classification. The authors compared their explanations to those generated by 3D-CAM and 3D-GRAD-CAM (Yang et al., 2018) methods. Jin et al. (2019) observed that these two 3D methods led to a substantial drop in model performance when classifying subjects for Alzheimer's Disease (AD) by the extra calculations needed to generate the heatmaps. By introducing the attention method, the authors obtained a 3D attention map for each testing sample and were able to identify the significance of brain regions related to changes in gray matter for AD classification. Our visualization technique may not be comparable to Jin et al. (2019), but the application of visualization techniques to medical imaging holds promise for making deep learning models interpretable.

## 5. Conclusion

We introduce a novel approach for the investigation of neural patterns in task-based fMRI that allow for the classification of dyslexic readers and typical readers. While deep learning classifiers provide accurate identification of dyslexic readers vs. typical readers based solely on their brain activation, such models are often hard to interpret. In this context, our main contribution is a visualization technique of the features that lead to specific classifications, which allows neuroscience domain experts to interpret the resulting models. Visual explanations of deep learning models allows us to compare regions instrumental to the classification with the latest neuroscientific evidence about dyslexia and the brain. The left occipital and inferior parietal regions that discriminated among groups are part of brain networks associated with phonological and lexical (word-level) processes in reading in different languages (Paulesu et al., 2001). Other regions reported in our visualization are also associated with reading and reading disorders (i.e., Tables 4, 5). More activation of anterior right-hemisphere prefrontal regions (e.g., right pars triangularis) are associated with dyslexia and possible compensatory mechanisms (Vellutino et al., 2004; Shaywitz and Shaywitz, 2005).

Feature visualization techniques and visual explanations for deep learning models are a novel research area, and applying these techniques to neuroimaging data has the potential to help research in neuroscience. Our work offers encouraging results, since the brain areas identified by the visual explanations are consistent with neuroscientific knowledge about the neural correlates of dyslexia. There are a number of ways in which we could extend the present work. The deep learning classification models can be applied to publicly available, large fMRI or MRI datasets to investigate the areas that are instrumental for identification of, for example, autism spectrum disorder. Moreover, other visualization techniques can be applied to provide a qualitative comparison among techniques when used to illustrate machine learning and deep learning studies of brain imaging.

## Data Availability Statement

The code to reproduce our experiments is available at GitHub (https://github.com/lauratomaz/VisualExplanations). Data was provided by BraIns (Brain Institute of Rio Grande do Sul - Brazil) initiative to establish a brain database of dyslexic readers of Brazilian Portuguese. This data can be found here: https://inscer.pucrs.br/br/neuroimagem-da-cognicao-humana.

## Ethics Statement

The studies involving human participants were reviewed and approved by PUCRS Ethics Committee. Written informed consent to participate in this study was provided by the participants' legal guardian/next of kin.

## Author Contributions

LT designed the study, implemented the framework, and ran experiments. FM and DR supervised the implementation and engineering of the work. AB and NE helped interpreting the findings and provided neuroimaging-related insights. All authors contributed to writing the manuscript.

## Conflict of Interest

The authors declare that the research was conducted in the absence of any commercial or financial relationships that could be construed as a potential conflict of interest.

## Publisher's Note

All claims expressed in this article are solely those of the authors and do not necessarily represent those of their affiliated organizations, or those of the publisher, the editors and the reviewers. Any product that may be evaluated in this article, or claim that may be made by its manufacturer, is not guaranteed or endorsed by the publisher.
