# Supplementary material for: Visual Explanation for Identification of the Brain Bases for Developmental Dyslexia on fMRI Data
Source: Front Comput Neurosci. 2021 Sep 9;15:594659. doi: 10.3389/fncom.2021.594659 (PMC8458961; doi:10.3389/fncom.2021.594659)
Supplement: Supplementary file 1 [file Data_Sheet_1.PDF]

Supplementary Material

1 PARTICIPANTS

The socioeconomic status of dyslexic readers and typical readers were, on average, levels B2 (lower-middle class) in Brazil (DYS 26.4 ± 6.5; TYP 24.1 ± 4.9); there were no significant SES differences between the groups. The IQ was determined for all participants in the two groups (Wechsler Abbreviated Scale of Intelligence). There were no significant IQ differences between the groups (dyslexic readers 107.85 ± 26.6, range $88–144$; typical readers 102.73 ± 15.37, range $71–127$) (Buchweitz et al., 2019).

2 INSTRUMENTS

The following tests were applied in the reading evaluations: word and pseudoword reading task (Salles et al., 2013); reading fluency (silently and out-loud) and comprehension test (Saraiva et al., 2009). These tests are validated for children learning to read in Brazilian Portuguese.

3 SUPPLEMENTARY FIGURES

The visualization showed regions that were instrumental to the classification (Figure S1). The regions included: (i) the left occipital lobe (including the left fusiform gyrus) with a high classification mapping for dyslexic readers; and (ii) the anterior cingulate cortex (ACC) with a high classification mapping for typical readers. Figure S$1a$ illustrates the classification mapping for dyslexia in left occipitotemporal region corroborates brain imaging findings that show functional alterations in this region associated with dyslexia and poor reading (Shaywitz et al., 2002; Martin et al., 2015; Pugh et al., 1996). Figure S$1b$ illustrates high classification mappings found in ACC for controls; activation of the ACC is usually associated with strategic control and attention processes (Chein and Schneider, 2005; Bush et al., 1999). More ACC activation has been found in association with increased working memory and attention workload in early good readers, and also in poor readers who benefited most from reading remediation in (Shaywitz et al., 2002; Buchweitz et al., 2019; Shaywitz et al., 2003).

We carried out a t-test analysis to show the traditional comparison of activation differences between groups. The t-test analysis (dyslexic readers versus typical readers) showed (Figure S4) more activation of the right insula in the word-reading task for the typical readers relative to dyslexic readers.

REFERENCES

Buchweitz, A., Costa, A. C., Toazza, R., de Moraes, A. B., Cara, V. M., Esper, N. B., et al. (2019). Decoupling of the occipitotemporal cortex and the brain's default-mode network in dyslexia and a role for the cingulate cortex in good readers: A brain imaging study of brazilian children. *Developmental neuropsychology* 44, 146–157. doi:10.1080/87565641.2017.1292516

Bush, G., Frazier, J. A., Rauch, S. L., Seidman, L. J., Whalen, P. J., Jenike, M. A., et al. (1999). Anterior cingulate cortex dysfunction in attention-deficit/hyperactivity disorder revealed by fMRI and the counting stroop. *Biological Psychiatry* 45, 1542–1552. doi:10.1016/S0006-3223(99)00083-9

Chein, J. M. and Schneider, W. (2005). Neuroimaging studies of practice-related change: fMRI and meta-analytic evidence of a domain-general control network for learning. *Brain research. Cognitive brain research* 25, 607–23. doi:10.1016/j.cogbrainres.2005.08.013

Cox, R. W. (1996). AFNI: software for analysis and visualization of functional magnetic resonance neuroimages. *Computers and biomedical research, an international journal* 29, 162–73

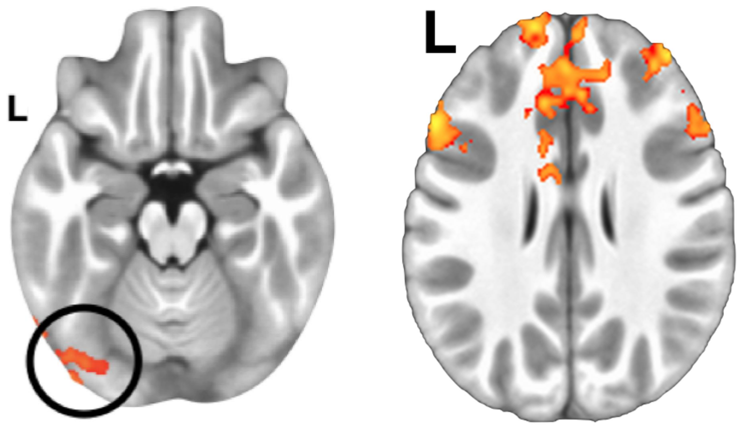

a **b**

Figure S1. Example of visual explanation for Dyslexic reader (a) and Typical reader (b) subjects respectively. The image a activation highlights left occipitotemporal region (slice at $z = 28$). The image b activation highlights ACC (slice at $z = -12$. AFNI (Cox, 1996) images showing brain activation from GRAD-CAM.

Martin, A., Schurz, M., Kronbichler, M., and Richlan, F. (2015). Reading in the brain of children and adults: a meta-analysis of 40 functional magnetic resonance imaging studies. *Human brain mapping* 36, 1963–81. doi:10.1002/hbm.22749

Pugh, K. R., Shaywitz, B. A., Shaywitz, S. E., Constable, R. T., Skudlarski, P., Fulbright, R. K., et al. (1996). Cerebral organization of component processes in reading. *Brain* 119, 1221–1238

Salles, J. F. d., Piccolo, L. d. R., Zamo, R. d. S., and Toazza, R. (2013). Normas de desempenho em tarefa de leitura de palavras/pseudopalavras isoladas (lpi) para crianças de 1º ano a 7º ano. *Estudos e Pesquisas em Psicologia* 13, 397–419

Saraiva, R. A., Moojen, S. M. P., and Munarski, R. (2009). Avaliação da compreensão leitora de textos expositivos: para fonoaudiólogos e psicopedagogos. *São Paulo: Casa do Psicólogo*

Shaywitz, B. A., Shaywitz, S. E., Pugh, K. R., Mencl, W. E., Fulbright, R. K., Skudlarski, P., et al. (2002). Disruption of posterior brain systems for reading in children with developmental dyslexia. *Biological psychiatry* 52, 101–110

Shaywitz, S. E., Shaywitz, B. A., Fulbright, R. K., Skudlarski, P., Mencl, W., Constable, R., et al. (2003). Neural systems for compensation and persistence: young adult outcome of childhood reading disability. *Biological Psychiatry* 54, 25–33. doi:10.1016/S0006-3223(02)01836-X

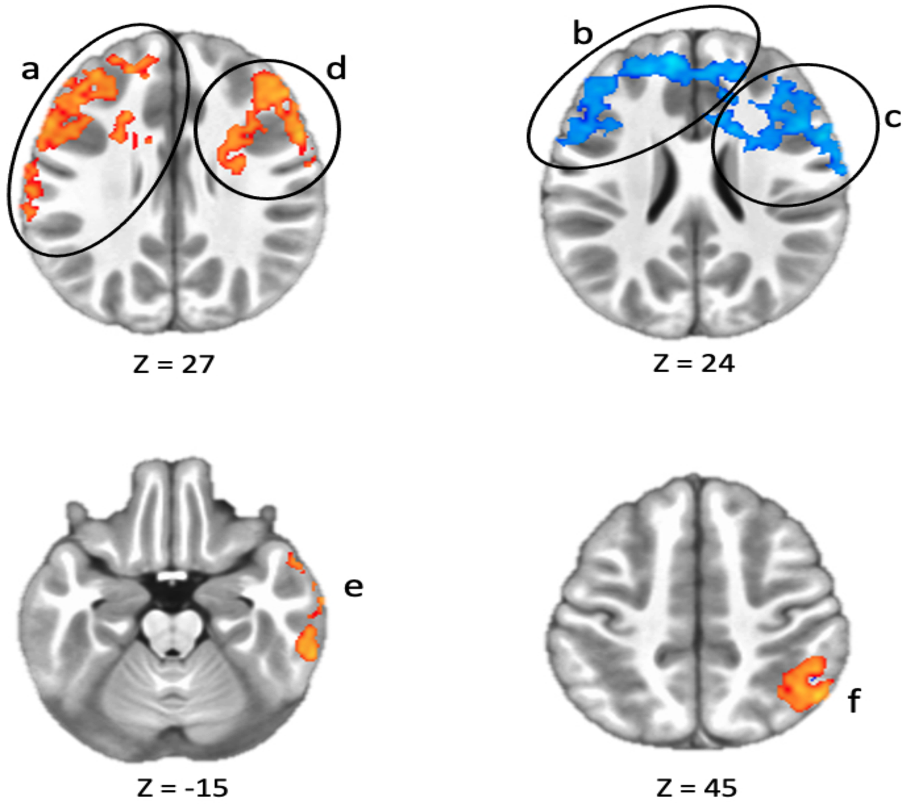

Figure S2. Example of visual explanation for Dyslexic readers subjects. Circle highlights instrumental brain regions for Dyslexic identification summarized in Table 4 in our paper. The left side of the images represent the left side of the brain. Images a, b, c, d, e, f depict a slice at $z = 27, 24, -15, 45$. AFNI (Cox, 1996) images showing brain activation from GRAD-CAM.

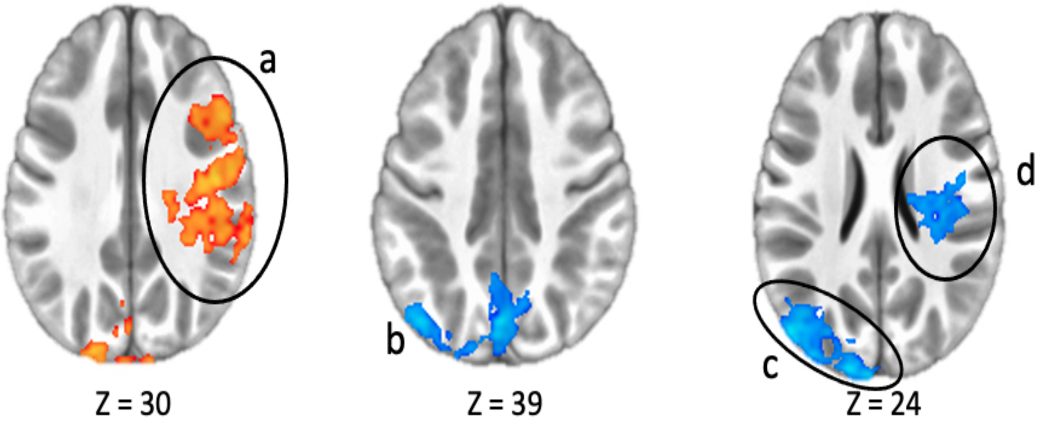

Figure S3. Example of visual explanation for Typical readers subjects. Circle highlights instrumental brain regions for Typical readers identification summarized in Table 5 in our paper. The left side of the images represent the left side of the brain. Image a, b, c depict slices at $z = 30, 39, 24$. AFNI (Cox, 1996) images showing brain activation from GRAD-CAM.

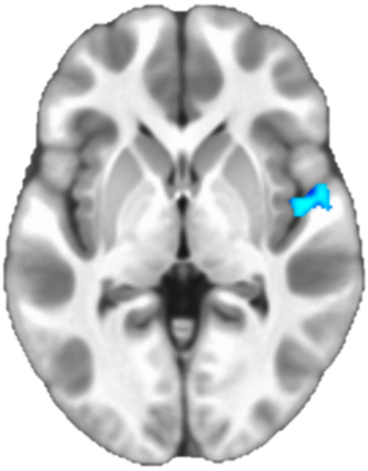

Figure S4. T-test analysis result for group comparisons (Typical Readers $>$ Dyslexic Readers). Results showed less activation on the right insula for dyslexic readers relative to typical readers during the word-reading task. Clusters significant at $\boldsymbol{p} < 0.05$, corrected for multiple comparisons (31 voxels; MNI coordinates $x = 52$; $y = -3$; $z = 3$).

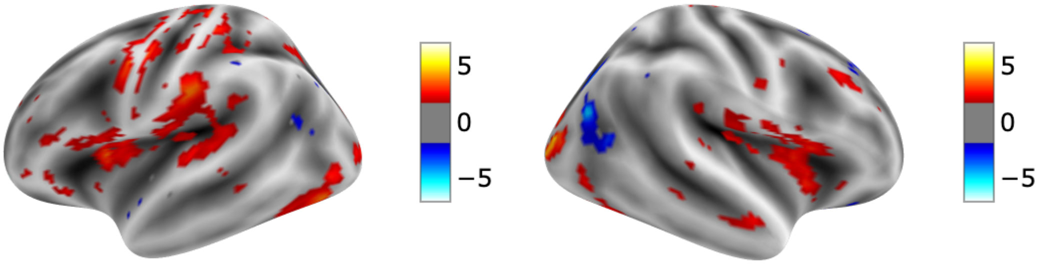

a **b**

Figure S5. T-test analysis results for typical readers during word-reading task. The left side of the images represents the left side of the brain.

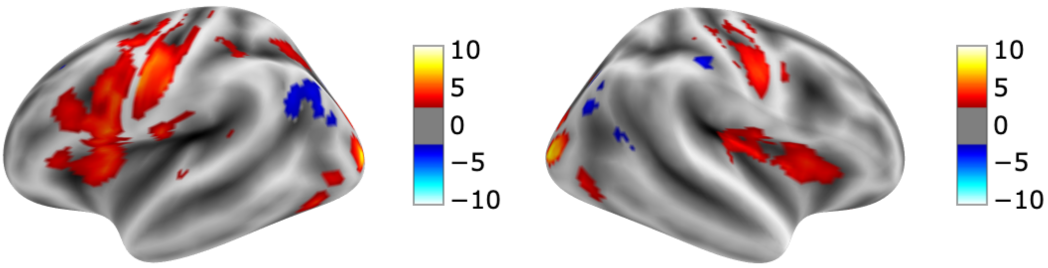

a **b**

Figure S6. T-test analysis results for dyslexic readers during word-reading task. The left side of the images represents the left side of the brain.
